# The influence of immediate occlusal loading on micro/nano-structure of peri-implant jaw bone in rats

**DOI:** 10.1186/s40729-024-00538-x

**Published:** 2024-05-09

**Authors:** Hiroaki Yorioka, Yuto Otsu, Ryu Suzuki, Satoru Matsunaga, Takayoshi Nakano, Shinichi Abe, Hodaka Sasaki

**Affiliations:** 1 Department of Oral and Maxillofacial Implantology, Tokyo Dental College, 2-9-18 Kandamisaki-cho, Chiyoda-ku, 101-0061 Tokyo, Japan; 2 Oral Health Science Center, Tokyo Dental College, 2-9-18 Kandamisaki-cho, Chiyoda-ku, 101-0061 Tokyo, Japan; 3 Department of Anatomy, Tokyo Dental College, 2-9-18 Kandamisaki-cho, Chiyoda-ku, 101-0061 Tokyo, Japan; 4 Division of Materials and Manufacturing Science, Graduate School of Engineering, Osaka University, 2-1, Yamada-oka, 565-0871 Suita, Osaka Japan

**Keywords:** Immediate implant placement, Immediate loading, Bone quality, Biological apatite orientation, Collagen fiber bundle anisotropy

## Abstract

**Purpose:**

The objective of the present study was to ascertain the effect of immediate occlusal loading after implant placement on osseointegration and the micro/nanostructure of the surrounding bone.

**Methods:**

After extraction of a rat maxillary right second molar, an implant was placed immediately with initial fixation (2 N< ). The implants were placed to avoid occlusal loading due to mastication, and in the loaded group, a superstructure was fabricated and subjected to occlusal loading. Bone morphometry, collagen fiber anisotropy, and biological apatite (BAp) crystallite alignment were quantitatively evaluated in both groups after extraction and fixation of the jaw bone at Days 7 and 21 after surgery.

**Results:**

Osseointegration was observed in both groups. Bone morphometry showed significant differences in bone volume, trabecular number, trabecular thickness and bone mineral density (BMD) at Days 21 postoperatively (*P* < 0.05). A significant difference was also found in the trabecular separation at Days 7 postoperatively (*P* < 0.05). In the evaluation of collagen fiber anisotropy, collagen fiber bundles running differently from the existing bone were observed in both groups. In terms of BAp crystallite alignment, a specific structure was observed in the reconstructed new bone after implantation, and preferential orientation of BAp crystallite alignment was observed in the longitudinal direction of the implants in the Day 21 postoperative loaded group.

**Conclusion:**

When sufficient initial fixation is achieved at the time of dental implant placement, then the applied masticatory load may contribute to rapidly achieving not only bone volume, but also adequate bone quality after implant placement.

## Background

The success of dental implant treatment depends on achieving osseointegration, which is the state in which the living bone tissue and the implant body surface bearing the functional load are in direct contact [1, 2]. Osseointegration is achieved through a mechanism equivalent to wound healing of the bone tissue around the implant body [3, 4]. To achieve good osseointegration, a non-loading period of at least 3 months in the human mandible and at least 4 months in the maxilla is reportedly required, and it has generally been recommended that no occlusal load be imposed for a certain time after implant placement [5]. Recently, however, an increasing number of studies have reported the possibility of “early loading” within 3 months, or of “immediate loading” within 7 days, if the jawbone is in very good condition and robust primary stability can be achieved [6–12]. Nevertheless, bone volume screening of bone density and compact bone thickness at the prospective implant placement site, which is today mainly conducted by computed tomography (CT), is often insufficient by itself for local jawbone evaluation, and the timing of loading after implant placement is still the subject of vigorous debate, with clear evidence as yet unavailable [13–17]. To understand the mechanical function of the load on dental implants and the surrounding jawbone will require studies that accurately predict the effect of different loading conditions after implant placement on osseointegration and the surrounding jawbone at the micro/nanostructural levels.

Attempts to understand the mechanical function of the formed bone by means of bone quality analysis using materials engineering techniques are now attracting attention. After a National Institutes of Health (NIH) Consensus Conference in 2000, bone quality was added to bone density as an index for evaluating bone strength (NIH Consensus Development Panel on Osteoporosis Prevention, 2001). Bone quality, including bone microstructure and metabolic turnover, calcification level, and presence of microcracks, is a factor that affects the performance of bone [18]. The structural characteristics of bone on the micro/nano scale are known to be greatly affected by local stress [19]. Two particular bone quality factors are collagen fibers, which resist tensile stress, and biological apatite (BAp) crystallites, which resist compression stress, and recent studies have shown that a decrease in these bone quality factors causes bone to undergo marked degradation [20]. Studies of the bone quality factors of collagen fiber bundle anisotropy and BAp crystallite alignment may therefore enable the mechanical function of peri-implant bone to be predicted [21–25]. In other words, quantitative evaluation of bone morphology should be able to demonstrate that implant placement has improved bone volume. Quantitative evaluation of collagen fiber bundle anisotropy and BAp crystallite alignment should provide evidence of improved bone quality.

The objective of the present study was to ascertain the effect of immediate occlusal loading after implant placement on osseointegration and the micro/nanostructure of the surrounding bone by producing a rat implant occlusal loading model and conducting a quantitative evaluation of the bone structural properties and bone quality of peri-implant bone tissue in the presence and absence of immediate loading.

## Materials and methods

### Animals

The experimental animals used were 20, 5-week-old, male Wistar rats (*n* = 5 in each group), weighing approximately 140 g. These rats were randomly allocated to one of two groups, a control unloaded group, in which no superstructure was fitted after implant placement and no load was applied (*n* = 10), and a loaded group, in which a superstructure was fitted after implant placement and an immediate load was applied (*n* = 10).

**Fig. 1:**
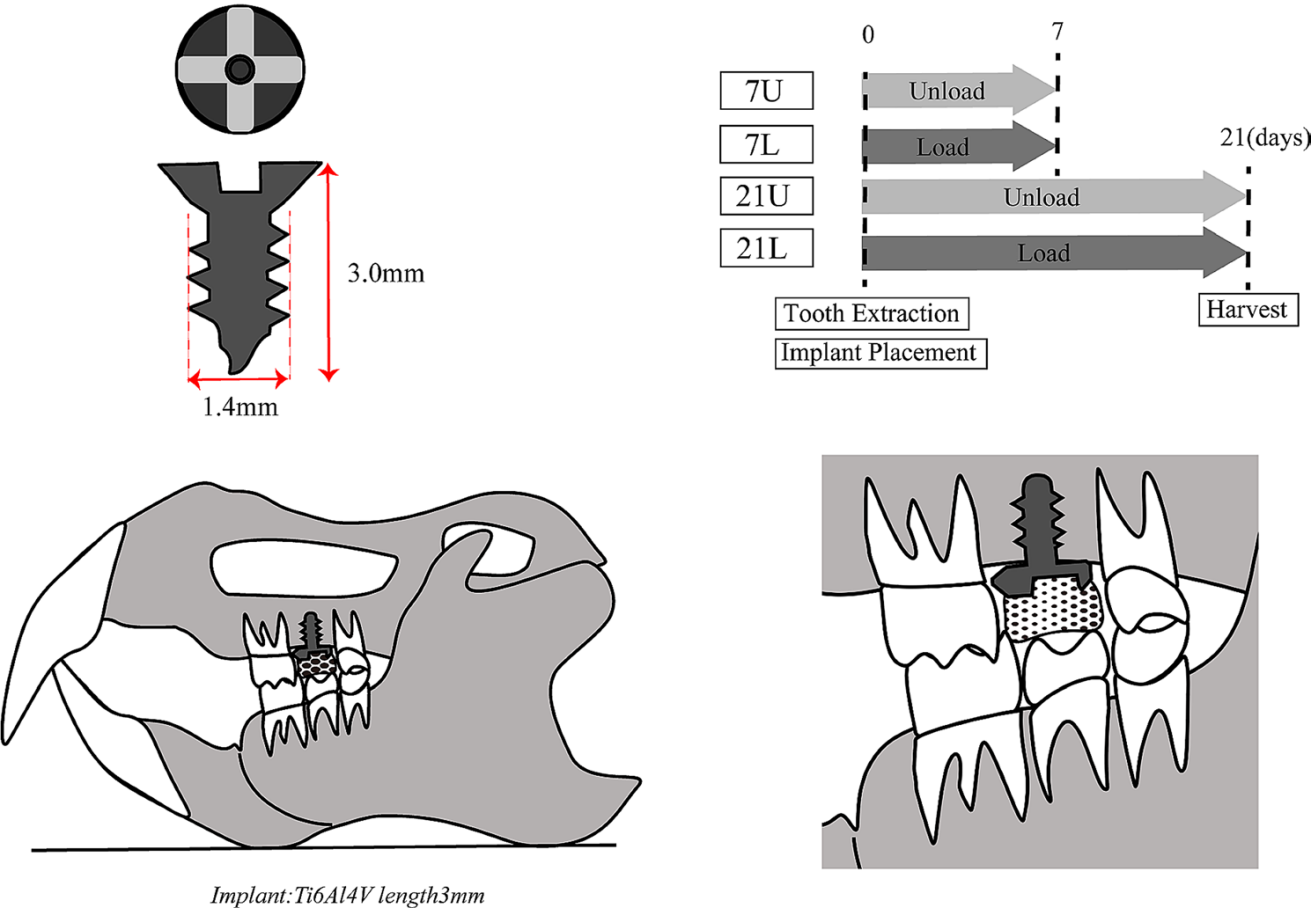
Experimental design. Implant details and placement location and experimental schedule

The rats were then kept under normal conditions until either Day 7 or Day 21, when they were euthanized, and their maxillae were harvested (Fig. 1). This animal experiment was conducted with the approval of the Tokyo Dental College Animal Experiment Committee (approval no. 223,302).

### Implant surgery

**Fig. 2:**
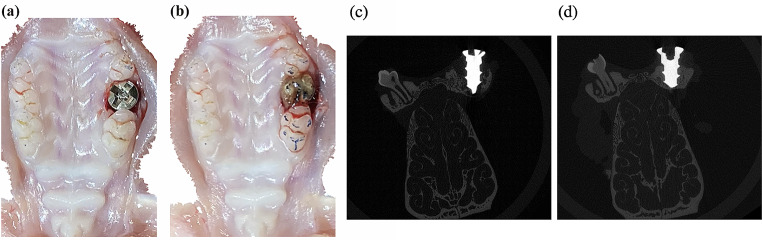
Intraoral findings and micro-CT images. (**a**) The maxillary right second molar is extracted, and a Ti-6A1-4 V implant (length 3 mm, diameter 1.4 mm, and 700 μm and 400 μm thread pitch and height) is placed. (**b**) After implant placement, the superstructure is built up with layers of resin until it touches the corresponding tooth in the lower jaw, an impression is taken with dental articulating paper, and this is compared with the pre-extraction impression to confirm the occlusal load. (**c**) Representative micro-CT image of Day 7. (**d**) Representative micro-CT image of Day 21

The implant placement procedure was conducted under general anesthesia induced by the intraperitoneal administration of a combination of three anesthetics (medetomidine hydrochloride 0.375 mg/kg, Nippon Zenyaku Kogyo Co., Ltd., Fukushima, Japan; midazolam 2.0 mg/kg, Sandoz K.K., Tokyo, Japan; and butorphanol tartrate 2.5 mg/kg Meiji Seika Pharma Co., Ltd., Tokyo, Japan). The occlusal status prior to extraction was confirmed by taking an impression with articulating paper. A probe was then used to extract the maxillary right second molar. A titanium alloy implant (Ti-6AL–4 V, length 3 mm, diameter 1.4 mm, and 700 μm and 400 μm thread pitch and height, Pro-seed Corporation, Tokyo, Japan) was placed in the extraction cavity by using a special screwdriver to achieve initial fixation with torque of at least 2 Ncm. Torque was confirmed to be at least 2 Ncm using a torque screwdriver (Nakamura Mfg. Co., Ltd., Tokyo, Japan). The implant head was positioned above the gingival margin to avoid direct occlusal loading and to avoid contact with adjacent teeth (Figs. 1 and 2a).

Resin (Sun Medical Co., Ltd., Shiga, Japan) was then used to create a superstructure.

The superstructure was gradually built up with layers of resin until it touched the corresponding tooth in the lower jaw, an impression was taken with dental articulating paper (Senjo Co., Ltd., Osaka, Japan), and this was compared with the pre-extraction impression to confirm the occlusal load (Figs. 1 and 2b). The superstructure was verified using dental floss (Ci Medical Co., Ltd., Ishikawa, Japan) to ensure that it was not fixed to both adjacent teeth.

### Specimen collection and micro-CT scanning

Immediately after harvesting, all samples were immersed in 40 ml of 10% neutral buffered formalin for 2 days. They were then transferred to 70% ethanol and scanned by micro-CT (µCT50; Scanco Medical AG, Wangen-Brüttisellen, Switzerland), and three-dimensional images were reconstructed using a volume rendering technique with TRI/3D-BON (Ratoc System Engineering Co., Ltd., Tokyo, Japan) and VGSTUDIO MAX 3.5 (Volume Graphics GmbH, Heidelberg, Germany) analysis software. The scanning conditions were as follows: tube voltage 90 kV, tube current 155 µA, image matrix 3400 × 3400, and slice thickness 2 μm. A 0.1 mm copper filter was inserted to address metal artifacts around the implant (Fig. 2c and d) [26].

### Histological evaluation

The specimens were subjected to graduated dehydration with ethanol, stained with Villanueva Osteochrome Bone Stain (Funakoshi Co., Ltd., Tokyo, Japan), and permeated with styrene monomer (Nissin EM Co., Ltd., Tokyo, Japan). They were then embedded in Rigolac® unsaturated polyester resin (Nissin EM Co., Ltd., Tokyo, Japan). The polymerized blocks were sliced in the XZ plane passing through the center of the implant body using a Leica SP 1600 saw microtome (Wetzlar, Germany) with a 300-µm-thick blade and polished with waterproof abrasive paper (#400, #800, and #1200) to prepare 100-µm-thick samples. These prepared specimens were examined under a general-purpose optical microscope (Axiophot2; Carl Zeiss, Oberkochen, Germany), and the volume of new bone was measured with the accompanying image software (Axiovision; Carl Zeiss).

### Bone morphometry

The region of interest was designated in the area of cancellous bone extending for 2.4 mm proximally and distally from the center of the implant (the implant body diameter is 1.4 mm). The bone volume [BV/TV (%)], trabecular number [Tb.N (mm)], trabecular thickness [Tb.Th (µm)], and trabecular separation [Tb.Sp (µm)] and BMD [BMD (mg/cc)] were calculated in three-dimensional structural analysis. The analysis was conducted with Scanco Medical software.

### Second harmonic generation (SHG) imaging

Second harmonic generation (SHG) images were acquired by using a multiphoton confocal microscopy system (LSM 880 Airy NLO; Carl Zeiss) with an excitation laser (Chameleon Vision II, wavelengths: 680–1080 nm; repetition rate: 80 MHz; pulse width: 140 fs; Coherent Inc., Santa Clara, CA, USA) and an objective lens (Plan-Apochromat 10×/0.8 M27; Carl Zeiss). The excitation wavelength for collagen fiber observation was 880 nm. The images thus obtained were used to measure the angle between the Z-axis and the negative direction of the collagen fiber bundles in an area measuring 200 μm × 200 μm centered on the implant central region (B and D). Since the collagen fiber bundles are drawn as curves, angle computation was conducted as a straight line connecting the ends of the curves in the area of observation. Collagen fiber bundle tracing and angle measurement were carried out using high-precision image analysis software (Imaris 8.4; Bitplane AG, Zürich, Switzerland). The variation in collagen fiber bundle angle was taken as an index of anisotropy.

### BAp crystallite alignment measurement

An optical curved imaging plate (IP) X-ray diffractometer (XRD: D/MAX PAPIDII-CMF; Rigaku Corporation, Tokyo, Japan) was used for the quantitative evaluation of BAp crystallite alignment. Measurements were made at the reference point in the 100-µm-thick, non-decalcified, polished samples, with the irradiation field positioned in the center of the thickness of the compact bone structure using the optical microscope fitted to the XRD (×0.6–4.8 magnification), and X-ray irradiation conducted so that the incident beam was a circular microbeam of diameter 100 μm. The reference axes were designated as previously described, with the X-axis running in the buccolingual direction, the Y-axis in the anterior-posterior direction, and the Z-axis in the implant long axis direction, and the specimens were positioned accordingly. In the implant neck region (A and E), the reference points were the points 100 μm from the implant surface in the X-axis direction.

**Fig. 3:**
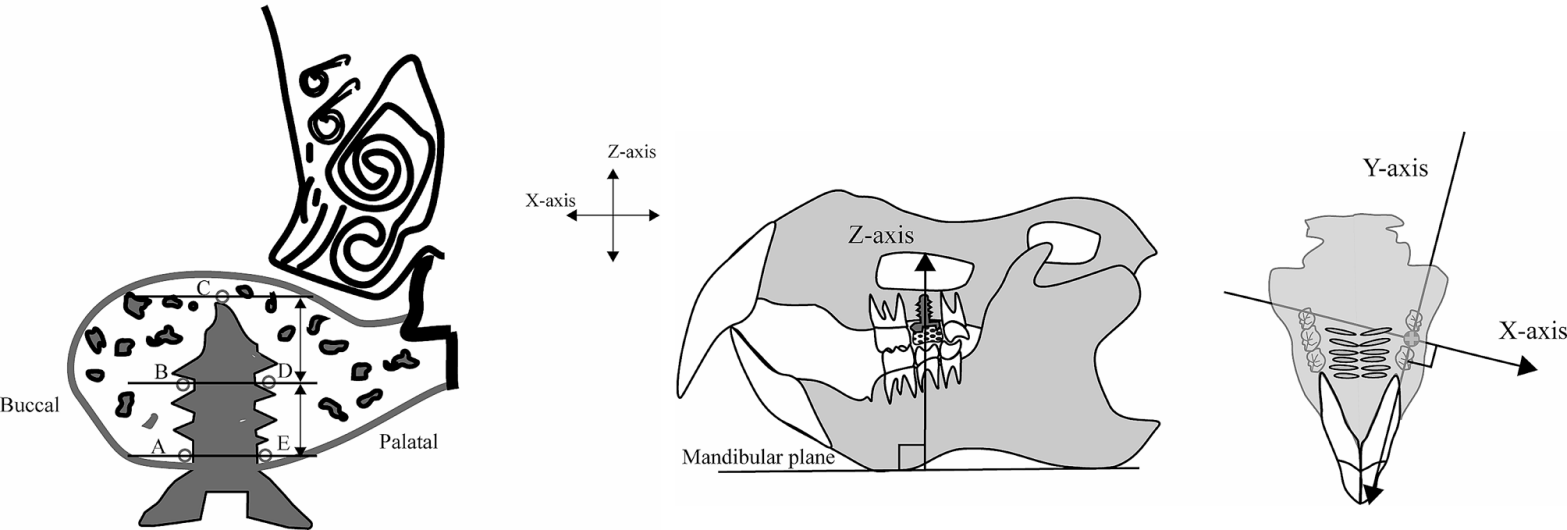
Designation of axes and setting the measurement point. Three axes are designated for measurements. The buccolingual direction is designated the X-axis, the mesiodistal direction the Y-axis, and the direction perpendicular to the occlusal plane (the long axis of the implant) the Z-axis. In the implant neck region (A and E), the reference points were the points 100 μm from the implant surface in the X-axis direction. The central parts of the long axis of the implant body were designated as B and D, and C was designated as the region at the apex of the implant body

The central parts of the long axis of the implant body were designated as B and D, and C was designated as the region at the apex of the implant body (Fig. 3). Measurements were made following the method described by Nakano et al. [21] with both the transmission optical system and the reflecting optical system, using Cu-Kα rays as the radiation source, with a tube voltage of 40 kV and tube current of 30 mA.

The diffraction X-ray beam was detected with a curved IP. Using 2D Date Processing Software (Rigaku Corporation, Tokyo, Japan), the X-ray intensity ratio of the two diffraction peaks in the (002) and (310) planes was calculated from the diffraction ring images generated on the IP by the diffraction X-ray beam.

### Statistical analysis

The results are expressed as mean values. GraphPad Prism version 6.0 (GraphPad Software, San Diego, CA, USA) was used for calculations and statistical analysis. After each measurement, t-test, one-way analysis of variance and Tukey’s multiple comparison test were conducted, and *p* < 0.05 was regarded as significant.

## Results

### Histological evaluation and bone morphometry

**Fig. 4:**
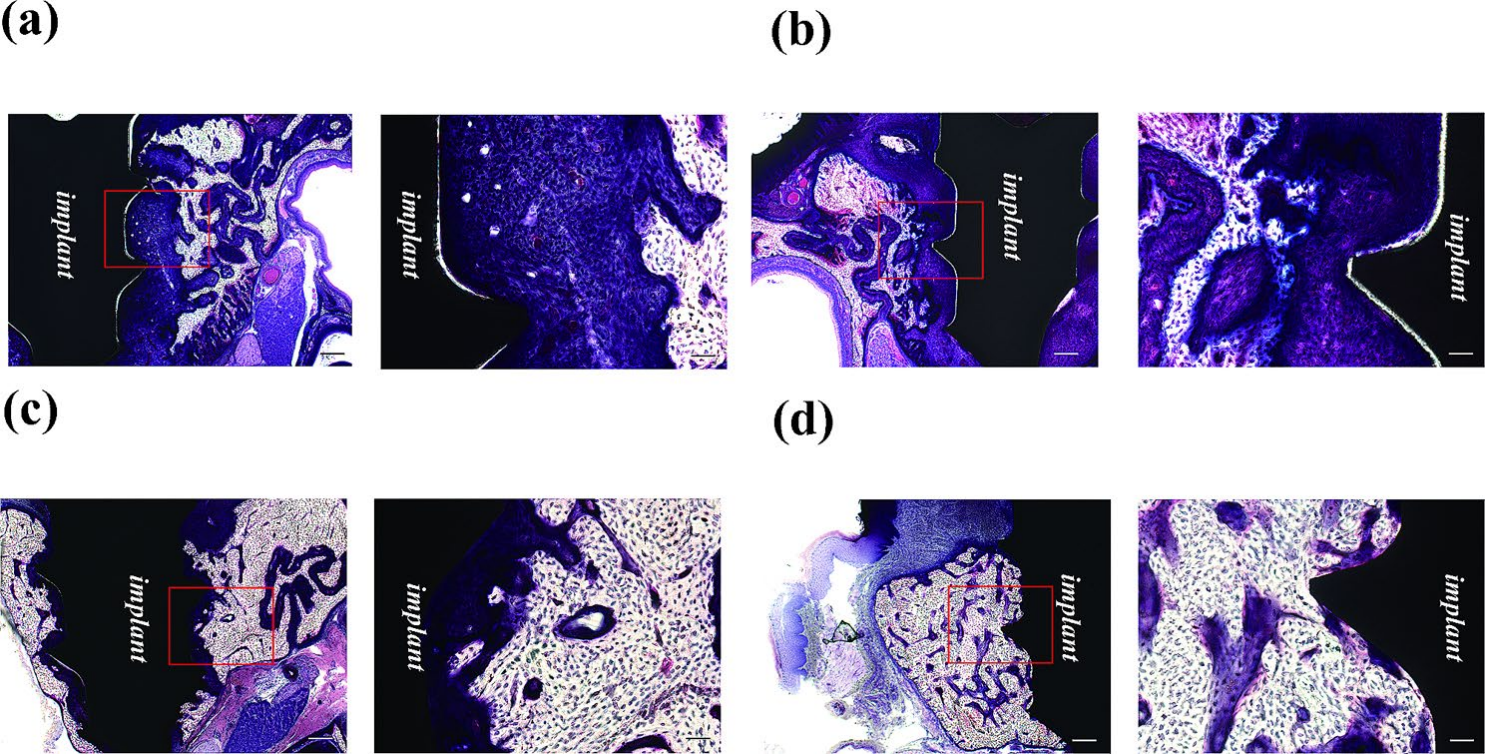
Histological explorations of peri-implant bone (Villanueva bone stain). (**a**) Unloaded group, Day 7, left 50× magnification, Bar: 200 μm, right 200× magnification. Bar: 50 μm (**b**) Unloaded group, Day 21, left 50× magnification, Bar: 200 μm, right 200× magnification. Bar: 50 μm (**c**) Loaded group, Day 7, left 50× magnification, Bar: 200 μm, right 200× magnification. Bar: 50 μm (**d**) Loaded group, Day 21, left 50× magnification, Bar: 200 μm, right 200× magnification. Bar: 50 μm

**Fig. 5:**
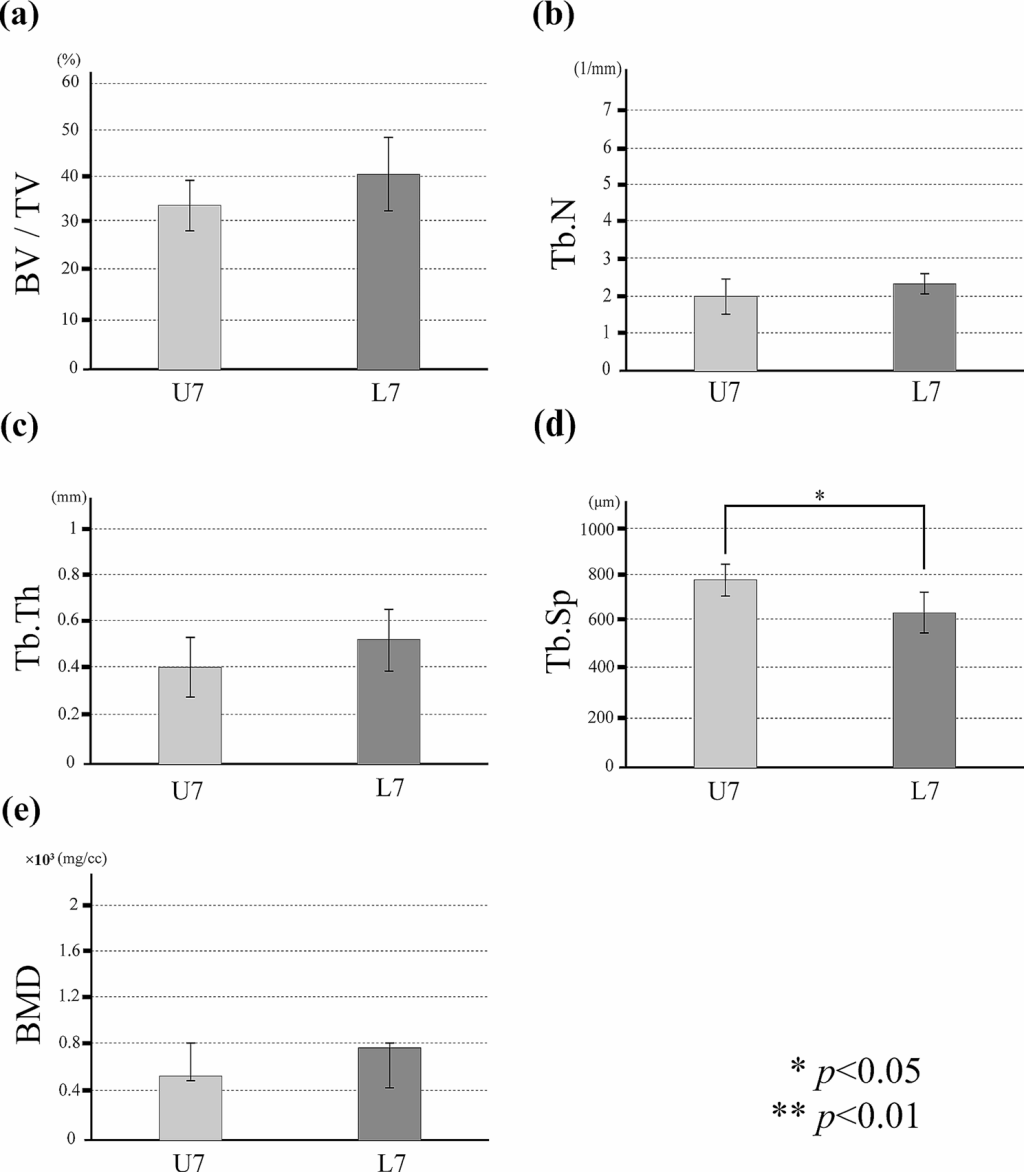
Evaluation of new peri-implant bone volume in the regions of interest. In the loaded and unloaded groups on Day 7, with the group on the horizontal axis, the vertical axis indicates (**a**) bone volume (%), (**b**) trabecular number (/mm), (**c**) trabecular thickness (µm), (**d**) trabecular separation (µm), and (**e**) bone mineral density (mg/cc)

**Fig. 6:**
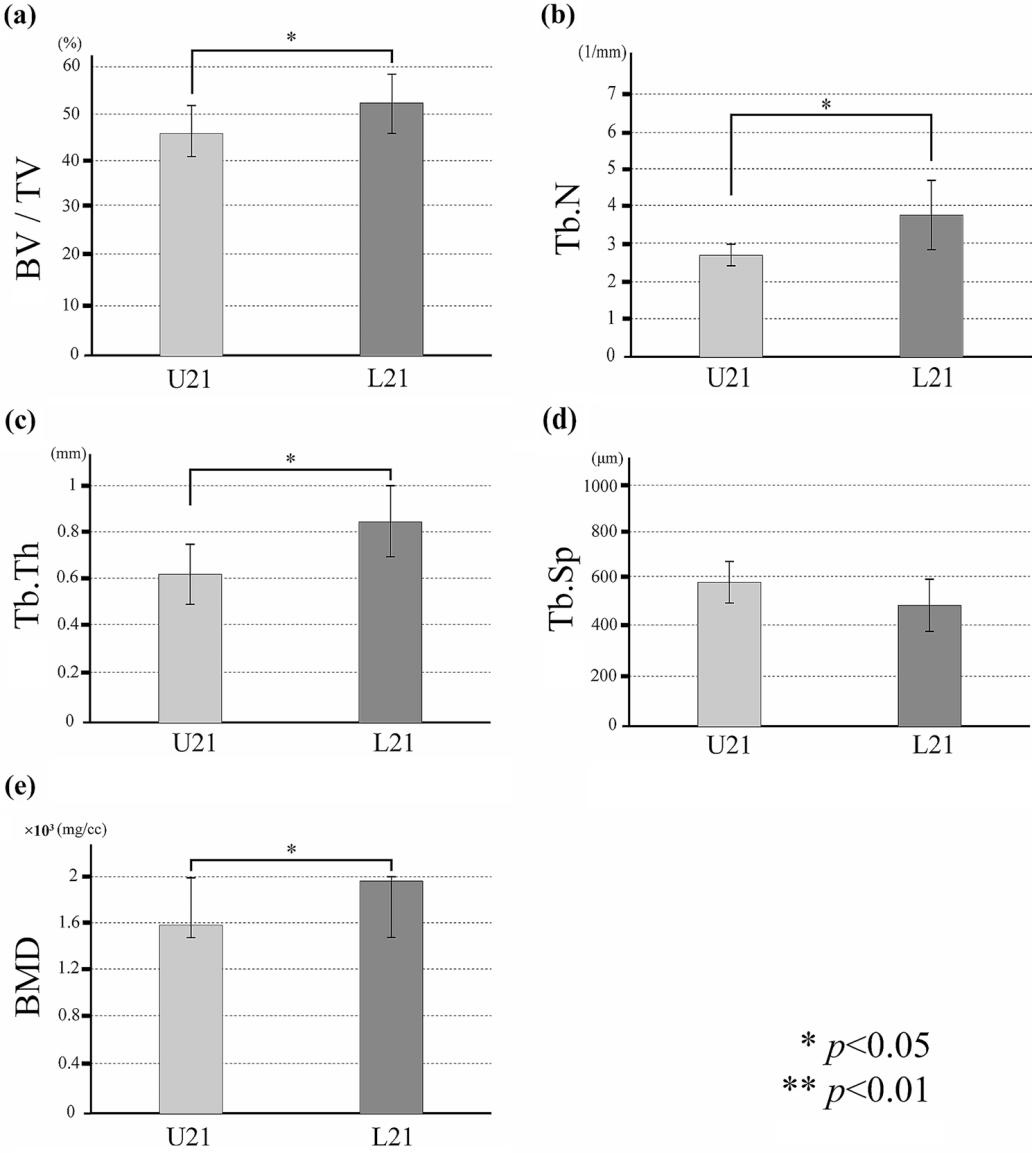
Evaluation of new peri-implant bone volume in the regions of interest. In the loaded and unloaded groups on Day 21, with the group on the horizontal axis, the vertical axis indicates (**a**) bone volume (%), (**b**) trabecular number (/mm), (**c**) trabecular thickness (µm), (**d**) trabecular separation (µm), and (**e**) bone mineral density (mg/cc)

Osseointegration between the implant and bone was observed in both groups. In all specimens, new bone with a compact bone-like structure was evident around the implants (Fig. 4a-d). BV/TV, Tb.N, Tb.Th and BMD were higher in the loaded group on both Day 7 and Day 21, and this difference was also significantly higher in the loaded group than in the unloaded group on Day 21 (*p* < 0.05) (Figs. 5 and 6). Tb.Sp tended to be lower in the loaded group at Day 7 and Day 21, with a significant difference at Day 7 (*p* < 0.05).

### SHG imaging

**Fig. 7:**
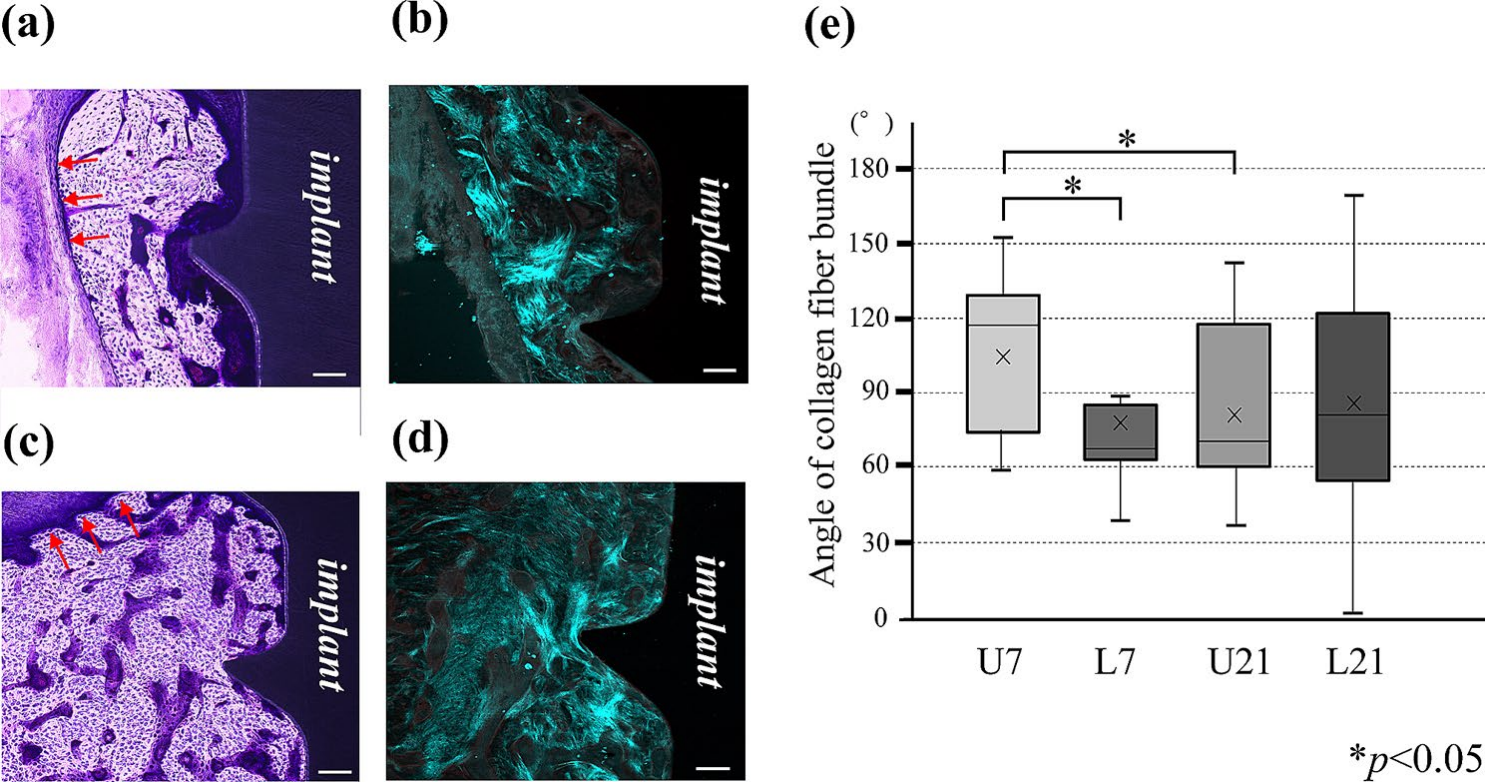
SHG imaging in the peri-implant area and evaluation of angle of collagen fiber bundle. (**a**): Optical microscopy of the peri-implant area in the unloaded group. (**b**): SHG imaging of the peri-implant area in the unloaded group. (**c**): Optical microscopy of the peri-implant area in the loaded group. (**d**): SHG imaging of the peri-implant area in the loaded group. a-d 100× magnification. Bar: 100 μm. (**e**): Box-and-whisker plot of the angle of the collagen fiber bundles with respect to the Z-axis. Red arrow: endosteum

SHG imaging showed collagen fiber bundles in the peri-implant bone with courses clearly different from those in the preexisting bone. In peri-implant bone, they tended to run in random directions, whereas in preexisting bone, they tended to run parallel to the endosteum (Fig. 7a-d). Numerous collagen fiber bundles were observed in the neighborhood of the endosteum, and most were running parallel to the endosteum. The courses of the collagen fibers in the peri-implant bone (B and D) were highly irregular (mean ± SD), with the angle with respect to the Z axis on Day 21 being 85.0º (±47.8º) in the loaded group and 79.6º (±30.6º) in the unloaded group. On Day 7, the angle with respect to the Z axis was 76.5º (±28.7º) in the loaded group and 105.1º (±30.6º) in the unloaded group. On Day 21, the collagen fiber bundle courses exhibited greater variation in angle in the loaded group than in the unloaded group, running irregularly in a variety of different directions (Fig. 7e).

### BAp crystallite alignment

**Fig. 8:**
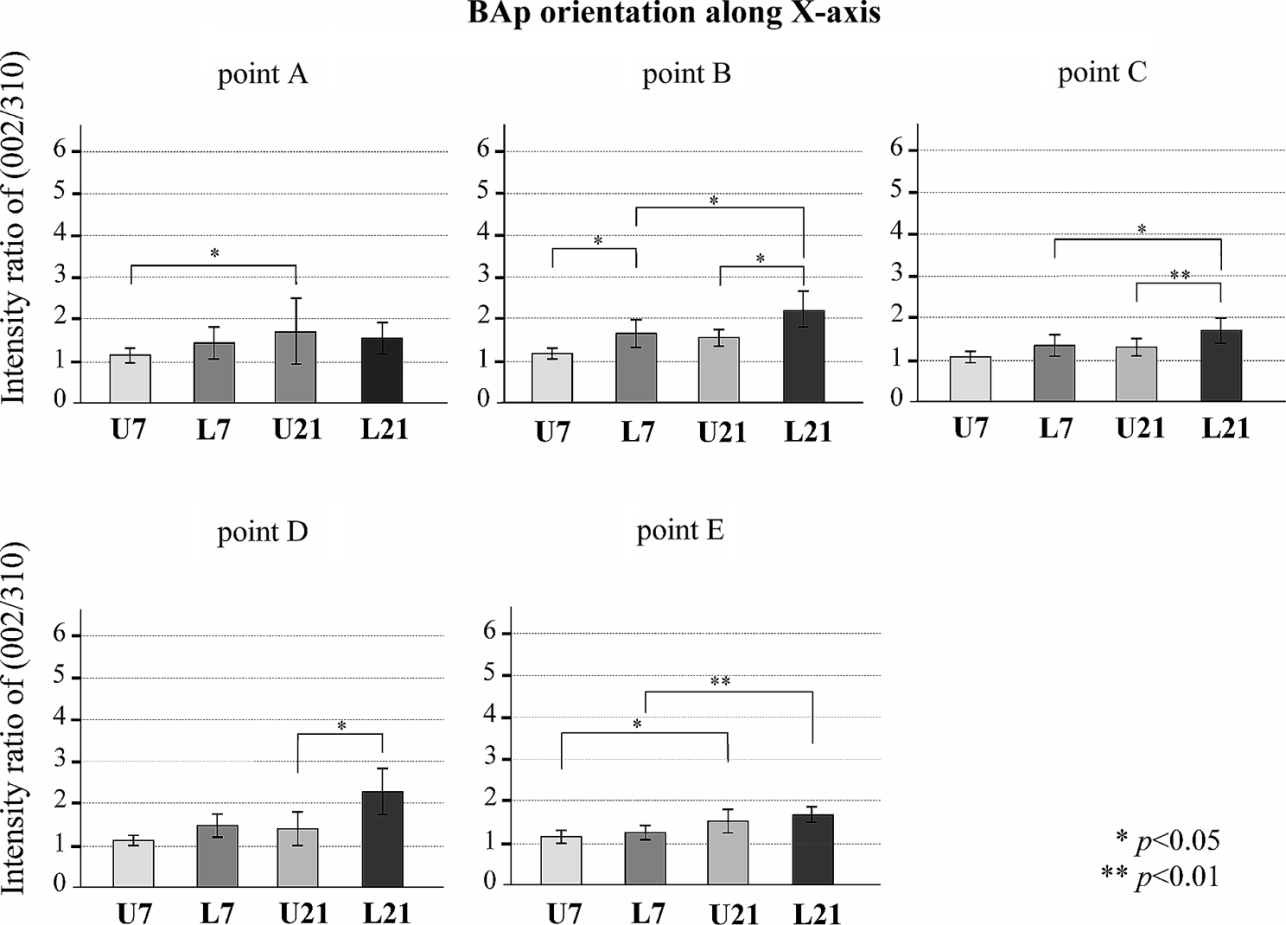
BAp crystallite alignment in the direction of the X-axis at each measurement point. Values are means ± the standard deviation

**Fig. 9:**
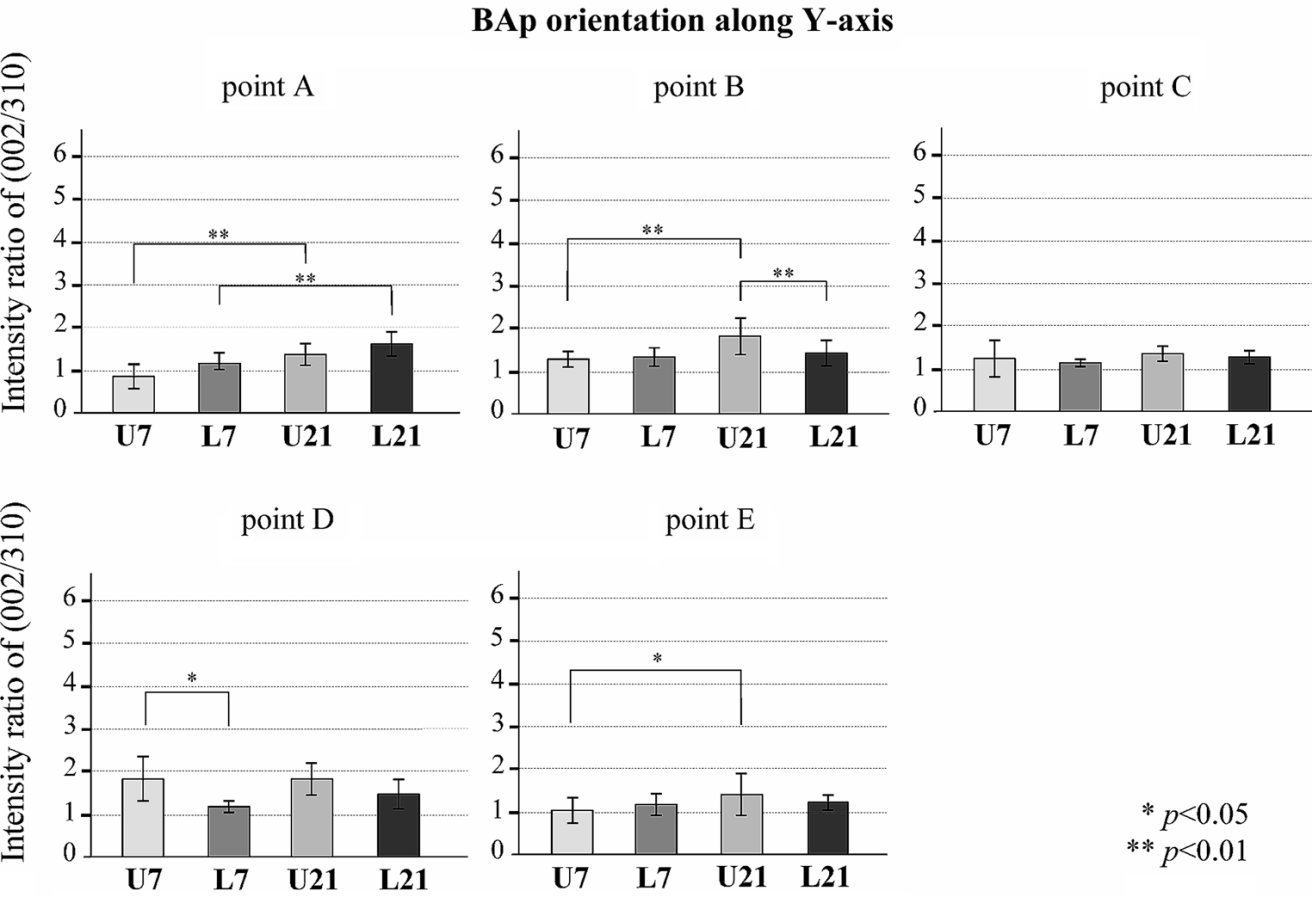
BAp crystallite alignment in the direction of the Y-axis at each measurement point. Values are means ± the standard deviation

**Fig. 10:**
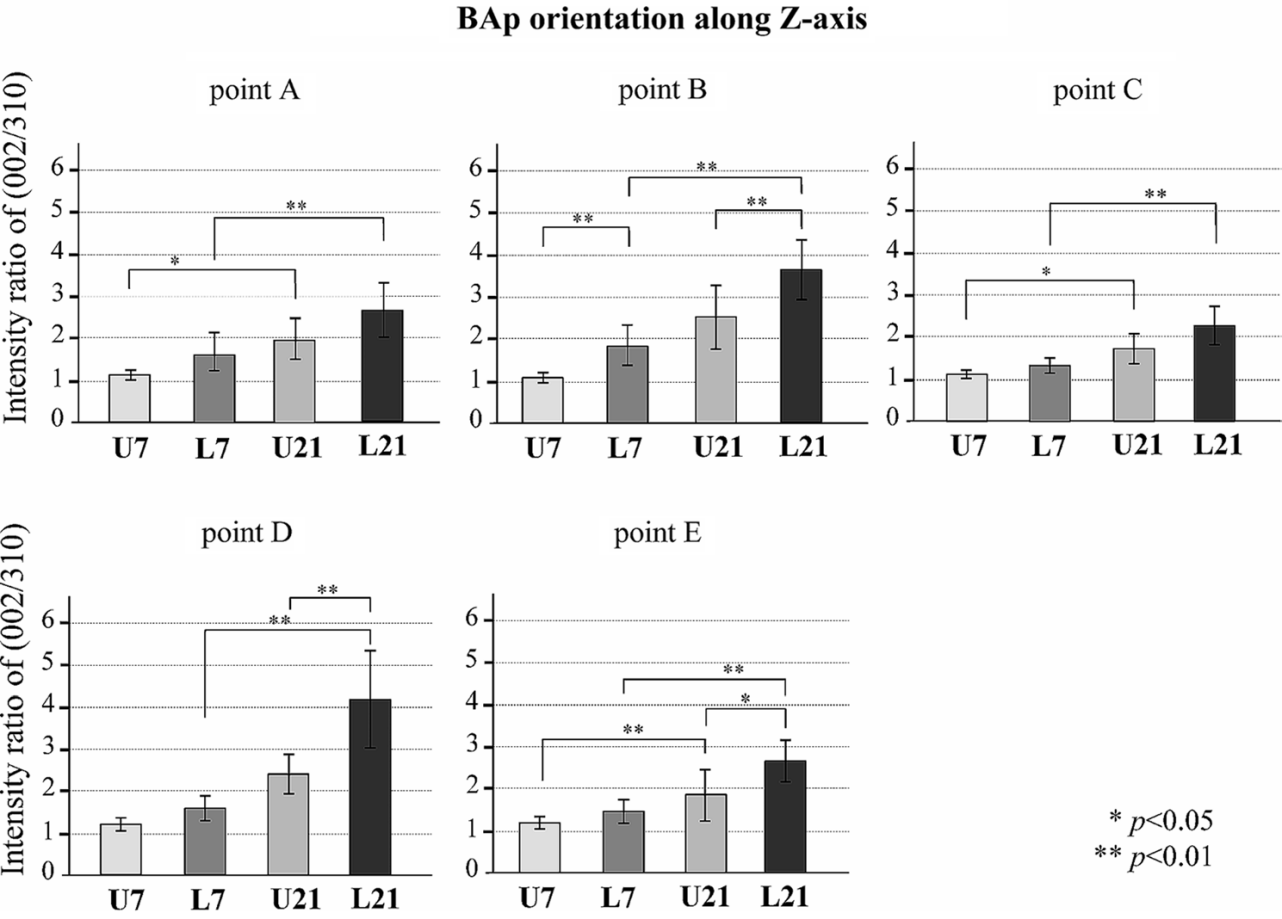
BAp crystallite alignment in the direction of the Z-axis at each measurement point. Values are means ± the standard deviation

Figures 8, 9 and 10 show the BAp crystallite alignments at five points measured in the X-axis and Y-axis with the transmission diffraction system and in the Z-axis with the reflecting diffraction system for the four groups (loaded and unloaded groups on Day 7 and Day 21). The X-ray diffraction intensity ratios in the x-axis direction were higher in the loaded group than in the unloaded group at points A, B, C, D (Fig. 8). However, there was no preferential alignment toward the X-axis direction at any of these points (*p* < 0.05). The X-ray diffraction intensity ratio in the y-axis direction tended to be higher in the loaded group at point A. There was no preferential alignment toward the Y-axis direction at any of these points (*p* < 0.05) (Fig. 9). The X-ray diffraction intensity in the Z-axis direction was the highest in the Day 21 loaded group and the lowest in the Day 7 unloaded group at all points. The loaded group also showed a significantly higher ratio of X-ray diffraction intensity in the Z-axis direction at Day 21 compared to Day 7 (*p* < 0.01). In addition, at points A, B, D and E, in the loaded group on Day 21, the c-axis of the BAp crystallites was preferentially aligned toward the Z-axis direction (Fig. 10).

## Discussion

When the periosteum, compact bone, and the blood vessels of the bone marrow are damaged by dental implant placement, after a hematoma has formed and the inflammatory response has occurred, osteogenic cells migrate to create bone granulation [27]. New blood vessels then invade this bone granulation, and within about two weeks, a fibrous callus is formed, which progresses to a bony callus. The new bone that subsequently forms is believed to achieve osseointegration by undergoing repeated bone remodeling as a result of mechanical stress [28]. The present histological observations showed that, in the loaded group, the bone tissue newly constructed around the implant exhibited a lamellar bone-like structure. The peri-implant bone in this study is almost consistent with the peri-implant bone morphology reported by Villar et al. suggesting that good osseointegration has been achieved through remodeling [29]. Al-Hamdan et al. observed a robust, compact bone-like structure in the vicinity of the implant-bone interface, and they reported that good osseointegration was achieved as a result [30].

In the unloaded group, on the other hand, the bone volume observed around the implant was lower than that in the loaded group. Romanos et al. reported that, in addition to binding at the implant-bone interface as a result of osseointegration, the mechanical function of the surrounding jawbone also plays an important biomechanical role in supporting dental implants [31]. Degidi et al. reported that the load imposed on the bone via the implant contributes to increasing peri-implant bone volume [32]. The present results also suggest that immediate loading after appropriate primary stability has been achieved may not only enable good osseointegration, but also contribute to the regeneration of the surrounding bone.

With respect to the load imposed on the implant, Duyck et al. reported that, although an appropriate load increases the rate of contact between bone and implant, overload causes peri-implant bone resorption [33]. The adverse effect of overloading on implants is widely known [34], since this is thought to cause bone resorption around the implant and to be one factor in reducing bone density [35]. Otsu et al. evaluated the bone surrounding implants placed in tail-suspended mice, and they found that minimal bone augmentation centered on the implant occurred around the implant even under extremely low loading conditions, but that the bone microstructure and bone quality were both diminished in comparison with those under normal loading [36]. In the present study, a comparison between bone morphometric results for cancellous bone in the loaded and unloaded groups showed that BV/TV, Tb.N, and Tb.Th were significantly higher in the loaded group, and that Tb.Sp was significantly lower in the unloaded group. This suggested that immediate loading after implant placement may have promoted bone resorption and bone formation, and led to the acquisition of bone microstructure and bone quality adapted to the new mechanical environment. Romanos et al. reported that immediate post-implant placement loading promoted bone formation around the implant, and that an unloaded period was disadvantageous to osseointegration, results that are not inconsistent with those of the present study [37].

With respect to bone quality, the results of SHG imaging analysis showed that collagen fiber bundles in the unloaded group ran almost parallel to the endosteum, whereas in the loaded group, the courses of the collagen fiber bundles in the new peri-implant bone were extremely irregular. Traini et al. reported that the collagen fiber bundles in compact bone of peri-implant jawbones were oriented perpendicular or at an angle to the direction of occlusal force [38]. Because the present study was conducted over a comparatively short period of time, the collagen fiber bundles may initially be arranged in an irregular configuration, and they may subsequently optimize themselves for their mechanical function through remodeling. With respect to BAp crystallite alignment, in a study in beagles, Odaka et al. found that peri-implant bone acquires a uniaxial preferential alignment to the occlusal direction as a result of remodeling [39]. Koresawa et al. also reported that the mechanical function imposed on dental implants results not only in osseointegration, but also in the active remodeling of peri-implant jawbone, contributing to the optimization of its micro/nanostructural characteristics [40]. In the present study, the loaded group exhibited significantly greater preferential alignment of the c-axis of BAp crystallites with respect to the long axis of the implant than did the unloaded group, which suggests that loading the implant may have helped achieve bone microstructure and bone quality adapted to the new mechanical environment with respect to occlusal force.

## Conclusions

When sufficient initial fixation is achieved at the time of dental implant placement, then the applied masticatory load may contribute to rapidly achieving not only bone volume, but also adequate bone quality after implant placement.

## Data Availability

Raw data were generated at Oral Health Science Center in Tokyo Dental College. Derived data supporting the findings of this study are available from the corresponding author [S. Matsunaga] on request.

## Abbreviations

BAp: Biological apatite
CT: Computed tomography
IP: Imaging plate
NIH: National Institutes of Health
SHG: Second harmonic generation
XRD: X-ray diffractometer
